# Nurses' Genomic Knowledge, Attitudes, and Perceived Organizational Support: A Comparative Secondary Analysis of Genetics and Genomics in Nursing Practice Survey Data

**DOI:** 10.1111/jnu.70107

**Published:** 2026-07-02

**Authors:** Ilknur Yesilcinar, Mari Laaksonen, Arja Halkoaho, Kathleen Calzone, Jacqueline Limoges

**Affiliations:** ^1^ University of Massachusetts Dartmouth, College of Nursing and Health Sciences Dartmouth Massachusetts USA; ^2^ Tampere University, Tampere University of Applied Sciences Tampere Finland; ^3^ Research Geneticist, Head‐Genomic Healthcare Section, Center for Cancer Research, Genetics Branch National Cancer Institute, National Institutes of Health Bethesda Maryland USA; ^4^ Athabasca University Athabasca Alberta Canada; ^5^ The Ontario Institute for Cancer Research Toronto Ontario Canada

**Keywords:** clinical competence, evidence‐based practice, genetic, genomic, nurses

## Abstract

**Aim:**

Identify similarities and differences in genomics‐informed nursing across five countries to support the development of actionable interventions that will facilitate the implementation of genomics in nursing practice and education globally.

**Introduction:**

The integration of genomics in nursing practice and education is a global challenge which can be addressed through effective policy and leadership that guide the integration of genomics into education and practice. In this study, cross‐country comparisons were conducted using secondary data derived from studies that employed the Genetics and Genomics in Nursing Practice Survey (GGNPS). This approach enabled us to analyze results accumulated over a 12‐year period and describe global trends in the development of genomic competencies within the nursing workforce. Identifying global trends in the development of genomic competencies and support within the nursing workforce could help unify efforts to strengthen cross‐country collaboration and address this long‐standing challenge.

**Methods:**

A comparative secondary analysis of the data from 10 studies that used the Genetics and Genomics in Nursing Practice Survey (GGNPS) and the Canadian Adaptation of the Genetics Genomics Nursing Practice Survey (GGNPS‐CA) was conducted between 2013 and 2025.

**Results:**

Over the past 12 years, the GGNPS/GGNPS‐CA survey results have remained largely unchanged. In all five countries utilizing the instrument, the majority of nurses recognized the importance of genomics. However, most nurses self‐rated their knowledge as poor, even with the average knowledge score of 8.62 out of 12. Nurses also consistently reported a lack of support from managers and senior staff for integrating genomics.

**Conclusion:**

Nurses were critical or uncertain of their knowledge, and they were not satisfied with the support they received. The similarity in results across the GGNPS/GGNPS‐CA surveys reinforces the global nature of nurses' challenges, underscoring the need for innovative educational approaches, strengthened leadership support, and coordinated global collaboration to address these issues.

**Clinical Relevance:**

Understanding the international nursing landscape in genomic education, competency, and practice serves as an evidence‐based foundation for cross‐country collaboration that can focus leadership, education, policies, and research to better support genomics‐informed nursing education and practice.

## 1 Introduction

Scientific advancements in genomics have introduced new opportunities and requirements for nurses across all five domains of practice (education, management, research, policy, and point of care) (Limoges, Halkoaho, et al. 2024; Zhao et al. 2022). Preparing the nursing workforce, which is the largest group of healthcare professionals and comprises approximately 30 million nurses and midwives (Boniol et al. 2022; World Health Organization 2025), is crucial for harnessing the full potential of genomic advancements and facilitating the integration of genomics into mainstream healthcare (Stark et al. 2019). To support this, an analysis of survey results from five countries which utilized the Global Genomics Nurses Practice Survey (GGNPS) (Calzone et al. 2013, 2014; Ceylan et al. 2025; Coleman et al. 2014; Gould and Seven 2022; Wang et al. 2023; Yeşilçinar et al. 2022a; Zhao et al. 2022) or its revised Canadian Adaptation of the Genetics Genomics Nursing Practice Survey (GGNPS‐CA) (Laaksonen et al. 2025; Limoges, Puddester, et al. 2024) was conducted. The aim was to identify similarities and differences in nursing and genomics across five countries to generate solutions that could enhance nurses' competencies and implementation of genomics.

## 2 Background

Genomics is an integral part of healthcare throughout the human lifespan and across multiple medical disciplines, supporting disease prevention, diagnosis, and treatment (American Society of Human Genetics, n.d.; Bilkey et al. 2019). There are, therefore, significant opportunities to advance nursing practice with genomics. Globally, resources exist to support genomics integration into nursing practice, such as curriculum and competency guidelines (Calzone, Jenkins, et al. 2018; Calzone et al. 2024), policy infrastructures (Chiu et al. 2024), and support for the known enablers to the integration of genomics into education and practice (Limoges, Halkoaho, et al. 2024). Education leaders have engaged in several educational experiments for undergraduate and postgraduate students (Genomics Education Programme, n.d.; Limoges, Halkoaho, et al. 2024; Smolander et al. 2025) and training programs to support the development of genomic literacy among healthcare professionals (Bokkers et al. 2022; Jenkins et al. 2015; van der Giessen et al. 2020). However, despite these efforts, McLaughlin et al. (2024) reported barriers to implementing genomic education. These included faculty uncertainty about students' baseline genetic knowledge and potential content overlap with other courses, schedule conflicts that hinder mastery, and the inherent complexity of genomics, which requires flexible delivery formats and sufficient time (McLaughlin et al. 2024). In a single‐institution project in the United States, the barriers nurses faced in obtaining genomic education included time constraints, limited availability of educational materials, and a perceived lack of clinical relevance (Hines‐Dowell et al. 2024).

Despite the increase in education and evidence‐based genomic clinical application, the integration of genomics‐informed nursing has yet to be fully optimized (Chiu et al. 2024; Dagan et al. 2021; Thomas et al. 2023) and the variation between countries is considerable (Calzone, Kirk, et al. 2018; Thomas et al. 2023). According to Thomas et al. (2023), there is increased interest in genomic nursing publications across more than 30 countries. The majority of these publications originate from the United States, the Netherlands, and United Kingdom, whereas studies conducted in other countries constitute only a small fraction of the overall literature (Thomas et al. 2023). Therefore, examining similarities and differences across countries using a common instrument is essential, as the existing body of research is unevenly distributed across national contexts, with some countries being underrepresented or lacking empirical data altogether.

Genomic competence has been examined from the perspective of nurses in many countries, and various instruments have been utilized in these assessments (Anderson et al. 2015; Cao et al. 2025; Laaksonen et al. 2023). According to literature reviews (Cao et al. 2025; Laaksonen et al. 2023), the most widely used survey instrument for assessment of competence of nurses for genomics is The Genetics and Genomics in Nursing Practice Survey (GGNPS), developed in the USA (Calzone et al. 2012). It is a quantitative assessment consisting of self‐evaluation and knowledge tests using multiple choice, dichotomous choice, and Likert scale questions (Calzone et al. 2012). It includes domains on competency/knowledge, confidence, attitudes/receptivity, decision/adoption, and social system (Calzone et al. 2012) based on the Diffusion of Innovation Theory (DOI) of Rogers (2003). The validity and reliability of the GGNPS was tested, confirming good face and content validity (Plavskin et al. 2019). Following reliability testing (Calzone et al. 2016), the instrument was revised, and subsequent research supported its theory‐based construct validity (Plavskin et al. 2023). The Turkish version of GGNPS also demonstrated its reliability (Yeşilçinar et al. 2022b) Over time, the GGNPS has been reviewed, leading to the incorporation of additional ethical considerations in GGNPS‐CA to address the social impact of genomics (Laaksonen et al. 2025; Limoges, Puddester, et al. 2024).

Measuring the nurses' knowledge in genomics is addressed in GGNPS and GGNPS‐CA (Calzone et al. 2012; Limoges, Puddester, et al. 2024). Knowledge has been recognized as a crucial first stage in the adoptive process according to the DOI (Rogers 2003). Both tools emphasize competence in general genomics and genetics rather than specialized expertise in a specific area of genomics. This aspect is important for understanding the competence level of genomics‐informed care among professionals who are not specialized in genetics and may work in any sector of healthcare. These instruments align with the idea of DOI that nurses should perceive the innovation as important and beneficial before it is adopted. Additionally, these two instruments consider the environment, which according to the DOI includes the organization and social system that influences individual actions (Rogers 2003). The organization and social system are addressed via the support of nursing management in both GGNPS (Zhao et al. 2022) and GGNPS‐CA (Limoges, Puddester, et al. 2024). Support from leaders and nursing management actions are important parts of implementing genomics (Calzone, Jenkins, et al. 2018; Tonkin et al. 2020b; Cao et al. 2025; Limoges et al. 2025). Enabling and encouraging nurses' participation in a variety of education is one of the responsibilities of nurse leaders (Calzone, Jenkins, et al. 2018; Kirk et al. 2011). Additionally, nurse leaders play a pivotal role in policy, practice, and regulation (Kirk et al. 2011) by raising awareness of the relevance of genetics, developing policies (Calzone, Jenkins, et al. 2018), and evaluating implementation progress (Tonkin et al. 2020a).

Given the robustness of the GGNPS and its alignment to the DOI, and that the GGNPS is the most commonly used survey instrument, this comparative analysis included studies utilizing the GGNPS and GGNPS‐CA. The studies were conducted worldwide, including the USA (Calzone et al. 2013, 2014; Coleman et al. 2014; Gould and Seven 2022), Canada (Limoges, Halkoaho, et al. 2024; Limoges, Puddester, et al. 2024), Turkey (Ceylan et al. 2025; Yeşilçinar et al. 2022a), China (Wang et al. 2023; Zhao et al. 2022), and Finland (Laaksonen et al. 2025). The comparative analysis focused on three areas: knowledge and confidence, attitudes and receptivity, and adoption and the social system. Within these focus areas, the comparative analysis examined nurses' level of genomic knowledge, perceptions of the importance of genomics, and the extent to which they felt supported by their managers and senior staff. Recommendations are proposed to accelerate genomics‐informed education and practice.

## 3 Methods

### 3.1 Aim

Identify similarities and differences in genomics‐informed nursing across five countries to support the development of actionable interventions that will facilitate the implementation of genomics in nursing practice and education globally.

Study questions:

1. What are the similarities and differences in nurses' genomic knowledge and confidence levels across the five countries over a 12‐year period?
2. What are the similarities and differences in nurses' attitudes toward genomics across the five countries over time?
3. What are the similarities and differences in organizational support across the five countries over time?

### 3.2 Primary Variables

In line with our study aim, the primary variables of the study are presented under the three areas: knowledge and confidence, attitudes and receptivity, and adoption and the social system that align with Roger's DOI Theory and GGNPS domains. In this study, the variables compared were identical across countries, except for a minor modification to one question in the Finnish adaptation (Laaksonen et al. 2025) (see Table 1). The variables are shown in Table 1.

**TABLE 1:** GGNPS and GGNPS‐CA variables, areas and answer options used for data extraction.

Areas	Primary variables of GGNPS/GGNPS‐CA for the study	Options
Knowledge and confidence	Knowledge Score Items^a^	P2‐2a, P2‐2b, P2‐2c, P2‐2d, P4‐1a, P4‐1b, P4‐1c, P4‐1d, P4‐1f, P4‐3b^b^, P5‐1, P5‐2
Knowledge and confidence	In describing your genetic/genomic knowledge, would you consider it to be	Excellent, Good, Poor
Attitudes and receptivity	How important do you think it is for the nurse to become more educated about the genetics of common diseases?	Very important/Somewhat important/Not very important/Not at all important/Neutral/Not sure/Don't know
Attitudes and receptivity	Please indicate whether you think each of the following would be a potential advantage of integrating the genetics of common diseases into your practice: Improved services to the patients Better adherence to clinical recommendations among patients	Advantage/No advantage
Adoption and the social system	In the past 3 months, has any patient initiated a discussion with you about genetics?	Yes, No, Don't know
Adoption and the social system	Do you intend to learn more about genetics?	Yes, No, Don't know
Adoption and the social system	Do you think your senior staff members see genetics as an important part of your role?	Yes, No, Don't know
Adoption and the social system	Do you think your senior staff members see genetics as an important part of their role?	Yes, No, Don't know
Attitudes and receptivity	Integrating genomics could lead to advantages such as improved service	Advantage, No advantage
Attitudes and receptivity	Integrating genomics could lead to advantages such as better adherence to clinical recommendations	Advantage, No advantage

^a^ See Data S1.

^b^ P4‐3b was changed in the Finnish version of GGNPS‐CA to “Do you think that genetic risk (e.g., as indicated by family history) has clinical relevance for mental health?”.

### 3.3 Ethical Considerations

The study was conducted in line with ethical principles and applicable guidelines for secondary data analysis. Most of the data analyzed in this study consist of previously published, publicly available, de‐identified secondary datasets that had already received ethical approval from the respective ethics committees in their original studies.

Ethics Committee approval was required for the collection of primary survey data from nurses, the results of which are reported for the first time in this publication (the Laaksonen dataset). This dataset received ethical approval from the Ethics Committee of The Tampere Region (statement number 46/2023).

### 3.4 Design

A comparative secondary analysis from nine previously published studies that used the GGNPS/GGNPS‐CA was conducted. In addition, one dataset from Finland was included. At the time of the data extraction, the Finnish study was unpublished; however, it met the inclusion criteria and followed the same methodology as the other studies. The study was later published in August 2025 (Laaksonen et al. 2025), however, the article focused on knowledge and ethical considerations and did not address all research questions examined in the present analysis. The authors had access to the portion of the Finnish dataset that was not included in the publication. These previously unpublished data are reported here for the first time. The inclusion of this dataset broadened the analysis by adding a country for which no prior evidence on practicing nurses' competence was available, thereby enhancing the international relevance of the study. The Finnish data were generated using the same methodology, ensuring comparability across studies and comprehensive coverage of all relevant DOI domains. A detailed description of the distribution of primary and secondary data is provided in Table 2. Thus, a total of 10 studies were included in the analysis.

**TABLE 2:** The main findings of the articles included in the review.

Data	Calzone et al. (2013), USA	Coleman et al. (2014), USA	Calzone et al. (2014), USA	Gould and Seven (2022), USA	Yeşilçinar et al. (2022a), Turkey	Zhao et al. (2022), China	Wang et al. (2023), China	Limoges, Puddester, et al. (2024), Canada	Ceylan et al. (2025)^a^, Turkey	Laaksonen et al. (2025), Finland
Knowledge score (max:12)	8.99	No data	8.8	9.55	9.36	8.30	7.35	8.59	Pre‐test: 3.31 Post‐test: 9.58	9.12
Self‐rated knowledge										
Poor	62.9%	50%	57.3%	41.7%	49.9%	61.9%	15.1%	73.0%	No data	78.6%
Good	25.1%	35%	No data	53.4%	45.9%	29.8%	60.8%	26.0%	No data	20.5%
Excellent	1.6%	2%	No data	6.8%	4.9%	8.3%	24.1%	1.0%	No data	0.4%
Becoming more educated about genomics is very or somewhat important	94.2%	98%	89%	61.5%	95%	73.4%	35%	91.7%	Pre‐test: 47.1% Post‐test: 100%	91.9%
Nurses perceived that managers saw genetics as important part of nurses' role	No data	No data	No data	31.1%	15.8%	36.4%	27.1%	12.5%	No data	15.0%^b^
Nurses perceived that senior staff saw genetics as an important part of their role	No data	No data	25.3%	35.8%	22.3%	36.4%	36.2%	15.5%	Pre‐test: 17.4% Post‐test: 92.6%	4.3%^b^
In the past 3 months, discussions initiated by patients about genomics	No data	72.0%	No data	46.6%	43.8%	No data	83.6%	19.0%	Pre‐test: 20.7% Post‐test: 24.0%	35.9%^b^
Nurses intend to learn more about genomics Did not know *No* *Yes*	No data No data 73.1%	No data No data 94.0%	No data No data 63.8%	No data No data 65.1%	17.1% 7.5% 74%	No data No data 57.3%	16% 27.8% 56.2%	39.0% 7.0% 53.0%	32.3 2.4% 65.3%	49.1%^b^ 30.8%^b^ 20.1%^b^
Integrating genomics could lead to advantages such as improved service	69.0%	68.0%	64.4%	87.7%	93.8%	87.5%	64.8%	92.3%	52.1%–99.2%	84.6%^b^
Integrating genomics could lead to advantages such as better adherence to clinical recommendations	84.8%	56.0%	50.0%	79.2%	92.5%	91.2%	64.8%	81.9%	Pre‐test: 54.5% Post‐test: 93.4%	81.2%^b^

^a^ Pre‐test and post‐test study results provided.

^b^ Primary, unpublished data for this article; Laaksonen dataset.

### 3.5 Search Strategy, Inclusion Criteria, and Data Extraction

The following keywords were used for the literature search: “genomic competencies” OR “genetics and genomics in nursing” OR “genetic competency” OR “genetics and genomics in nursing practice survey”. The literature was searched for articles published from January 2013 to January 2025 that used the GGNPS or GGNPS‐CA survey. This approach allowed us to examine the outcomes within a standardized, globally collected dataset. The literature search was conducted by one researcher using the CINAHL Complete database. The inclusion criteria (Table 3) were applied to ensure the selection of relevant studies. To be included in the study, at least half of the variables had to be available in the dataset: nurses' genomic knowledge scores, perceptions about their genomic knowledge, managerial and senior staff support, intent to learn more about genomics, and perceived advantages of integrating genomics into practice.

**TABLE 3:** Inclusion and exclusion criteria for literature review.

Inclusion criteria	Exclusion criteria
Original research studies using GGNPS/GGNPS‐CA Studies conducted with the participation of practicing nurses Studies provided data on nurses' knowledge scores, perceptions about their genomic knowledge, support from their managers and/or senior staff, intent to learn more about genomics, advantages of implementing genomics into practice Full‐text articles	Studies conducted by using GGNPS/GGNPS‐CA with only students or faculty, with no inclusion of practicing nurses Studies conducted using GGNPS/GGNPS‐CA but provided less than half of the variables that were set as primary variables Validation studies that consisted only of psychometric data Reviews Policy studies

The database search resulted in 213 papers. Duplicated papers were removed. After the title review, 191 papers were excluded solely based on their titles; subsequently, the abstracts and full texts were read to assess relevance. Editorials, reviews, and policy articles (*n* = 7) were excluded, and the two reviewers reviewed 15 articles. After identifying eligible studies, data extraction was conducted by two authors reviewing the study details (author(s), year, country, study design, and sample size), participant characteristics (practicing nurses), data collection tools (GGNPS/GGNPS‐CA), and the results with the focus on our primary variables. One author extracted data, and the second author independently checked the data to ensure accuracy and consistency. Discrepancies were resolved through discussion. The studies that did not include enough data regarding our predefined variables were excluded from the analysis (*n* = 6), leaving 9 published studies included in this analysis.

In addition, the dataset from Finland was included through manual selection. The division of the data into primary and secondary components is detailed in Table 2. This primary data is hereafter referred to as the Laaksonen dataset and secondary data as Laaksonen et al. (2025). Therefore, 10 articles were included in the analysis.

### 3.6 Data Analysis

In addition to the primary variables, the main characteristics of the studies—such as author(s), year, country, data collection period, data collection tools (GGNPS or GGNPS‐CA), total sample size, and the percentage of actively practicing nurses—were extracted into Microsoft Excel. The data were analyzed using a combination of descriptive analysis of the main characteristics and frequency analysis to summarize the primary variables of this study. Descriptive statistics such as percentages, mean, and maximum were used to summarize and compare data related to our primary variables. Mean and maximum values were provided for genomic knowledge scores only, and other variables were presented as percentages.

## 4 Results

### 4.1 Characteristics of Included Studies

The characteristics of the included articles are shown in Table 4. Studies were conducted in the USA (*n* = 4), Turkey (*n* = 2), and China (*n* = 2), Canada (*n* = 1), and Finland (*n* = 1). The time frame of data collection varied between March 2009 and December 2023. The respondents were nurses.

**TABLE 4:** Characteristics of the included studies.

Authors/Year	Country	The time frame of data collection	GGNPS/GGNPS‐CA	Total sample	Nurses actively taking care of patients
Calzone et al. (2013)	USA	October 2009 to January 2010	GGNPS	619	54.1%
Coleman et al. (2014)	USA	March 2009 to January 2010	GGNPS	392	42.0%
Calzone et al. (2014)	USA	July to October 2012	GGNPS	7798	73.1%
Gould and Seven (2022)	USA	March and May 2020	GGNPS	106	81.4%
Yeşilçinar et al. (2022a)	Turkey	January to April 2020	GGNPS	385	47.1%
Zhao et al. (2022)	China	November 2019 to January 2020	GGNPS	2118	100%
Wang et al. (2023)	China	January to March 2022	GGNPS	406	39.8%
Limoges, Puddester, et al. (2024)	Canada	November 2022 and February 2023	GGNPS‐CA	1012	72.3%
Ceylan et al. (2025)	Turkey	May 2023 and February 2024	GGNPS	121	86.0%
Laaksonen et al. (2025) (secondary)/Laaksonen dataset (primary)	Finland	October to December 2023	GGNPS‐CA	234	73.1%

The key findings were categorized into three groups: knowledge and confidence, attitudes and receptivity, and adoption and the social system. The variables and data utilized in this study are presented in detail in Table 2.

### 4.2 Genomic Knowledge and Confidence

**FIGURE 1:**
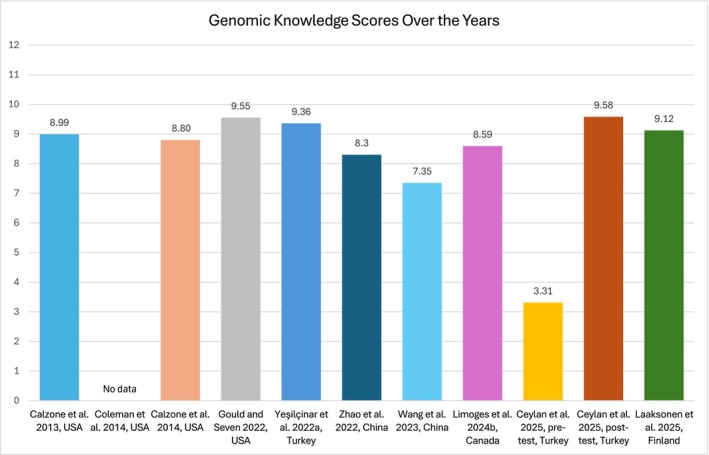
Genomic Knowledge Scores of nurses in different countries over the years.

Genomic knowledge in the articles included was measured with objective Knowledge Scores (KS) (see Data S1) and self‐rated knowledge assessment. Figure 1 shows the trends in nurses' genomic Knowledge Scores (KS) over the years. Although included studies categorized nurses' KS, only one study (Laaksonen et al. 2025) explicitly reported the rationale for classification into low, moderate, and good levels. Consequently, the present analysis focuses on comparing absolute values rather than categorical classifications used in the included studies. Nurses' KS ranged from a minimum of 3.31 (Ceylan et al. 2025) to a maximum of 9.55 (Gould and Seven 2022) out of 12. The first study by Calzone et al. (2014) reported 8.8 out of 12 points for genomic knowledge. In 2020, two different studies reported slightly higher KS in Turkey (9.36/12) (Yeşilçinar et al. 2022a) and in the USA (9.55/12) (Gould and Seven 2022). In 2022 and 2023, a slight decrease in KS were found in two studies conducted in China (8.30/12; 7.35/12) (Wang et al. 2023; Zhao et al. 2022). A recent study that compared the KS before and after education reported the lowest genomic knowledge levels at 3.31 before providing education to the nurses (Ceylan et al. 2025). Following WhatsApp‐based educational sessions, the nurses' KS increased dramatically to 9.58 (Ceylan et al. 2025). According to the most recent studies conducted between 2022 and 2025, the genomic KS were slightly lower in China, Canada, and Turkey (Ceylan et al. 2025; Limoges, Puddester, et al. 2024; Wang et al. 2023; Zhao et al. 2022) compared to the other studies that were done in Turkey, Finland, and the USA (Gould and Seven 2022; Laaksonen et al. 2025; Yeşilçinar et al. 2022a) as shown in Figure 1. One study did not provide data for mean KS (Coleman et al. 2014).

In the studies collected between 2009 and 2020 in the USA and Turkey (Calzone et al. 2013; Coleman et al. 2014; Gould and Seven 2022; Yeşilçinar et al. 2022a), approximately 50% of the nurses perceived that their self‐rated knowledge was poor (41.7%–50%). In the studies published after 2022 that were conducted in Finland, China, and Canada, nurses reported poor self‐rated knowledge, varying between 61.9% and 78.6% (Laaksonen et al. 2025; Limoges, Puddester, et al. 2024; Zhao et al. 2022) except Wang et al.'s study (2023) which was conducted in China where only 15.1% of the nurses perceived their genomic knowledge as poor (Wang et al. 2023). In Wang et al.'s study (2023), 60.8% of the nurses perceived their genomic knowledge as “good” and 24.1% as “excellent”; however, concurrently, the more objective KS were slightly lower compared with other studies. In another study conducted in 2013, 35% of the nurses perceived their genomic knowledge as “good” (Calzone et al. 2014), whereas it was 53.4% in Gould and Seven's (2022) study, which was conducted with the participation of nurse practitioners. (Yeşilçinar et al.'s study 2022a) 45.9% of the nurses reported that they perceived their genomic knowledge as ‘good’. More recent data, presented in studies from Finland and Canada, reported the most critical self‐rated knowledge perceptions. Only 20.5% (Laaksonen et al. 2025) and 26.0% (Limoges, Puddester, et al. 2024) self‐rated their knowledge to be at a good level.

### 4.3 Attitudes and Receptivity

The majority of nurses stated that it is very or somewhat important for nurses to become more educated about genomics. However, in Wang et al.'s study (2023) with a result of 35.0% and Ceylan et al.'s study (2025) 47.1% before the training session nurses indicated the same. Nurses' own intent to learn more about genomics varied. In most of the studies (9 out of 10), more than half of the nurses intended to learn more (data varied between 53.0% and 94.0%). In contrast to the other countries, only 20.1% of respondents in the Finnish study indicated an intention to learn more. However, nurses in Finland were not opposed further education, as 49.1% reported being uncertain about their intentions to engage in additional learning (Laaksonen dataset).

### 4.4 Adoption and Social System

In Calzone et al.'s study (2014), 72.0% of the nurses stated that they have had patient‐initiated discussions about genomics in the past 3 months. The studies conducted recently reported lower percentages varied between 19.0% and 46.6% (Ceylan et al. 2025; Gould and Seven 2022; Limoges, Puddester, et al. 2024; Yeşilçinar et al. 2022a, 2022b; Zhao et al. 2022; Laaksonen dataset), except for Wang et al. (2023), which reported higher results (83.6%).

In all the studies, more than half of the nurses (ranging from 52.1% to 99.2%) reported higher perceptions of the benefits of integrating genomics, which could lead to advantages such as improved healthcare. Similarly, a substantial proportion of nurses across the studies (ranging from 50.0% to 93.4%) agreed that integrating genomics could lead to better adherence to clinical recommendations.

In all studies, only a minority of nurses perceived that nurse managers regarded genetics as an important part of nurses' role. The responses ranged between 12.5% and 36.4%. Similar to these results, a minority of the nurses reported that their senior staff saw genetics as an important part of their own role. The responses ranged between 4.3% and 36.4% (Calzone et al. 2013; Ceylan et al. 2025; Coleman et al. 2014; Gould and Seven 2022; Limoges, Puddester, et al. 2024; Wang et al. 2023; Yeşilçinar et al. 2022a; Zhao et al. 2022; Laaksonen dataset). These numbers declined in the most recent studies included in this article and ranged between 4.5% and 17.4% (Ceylan et al. 2025; Limoges, Puddester, et al. 2024; Laaksonen dataset). In Ceylan et al.'s study (2025), the percentage increased to 92.6% after the training session.

## 5 Discussion

The findings describe knowledge and perception of genomics among nurses from five different countries over a 12‐year time span. The results illustrate that since the first included study (Calzone et al. 2013), nurses have consistently perceived genomics as important; however, a persistent gap is evident between their objectively measured genomic knowledge and their self‐rated understanding. Furthermore, nurses have conveyed receiving low support from their managers and senior staff. The following discussion contextualizes these findings within the broader literature and proposes solutions to address these persistent challenges that impact nurses from several countries.

### 5.1 Genomic Knowledge

In light of the uncertainty nurses expressed regarding their competence, the discussion highlights the need to develop targeted strategies to enhance nurses' confidence. Nurses in eight of the 10 studies perceived their subjective genomic understanding levels as low.

Only two studies, one in China (Wang et al. 2023), and one conducted in the USA (Gould and Seven 2022), reported that the majority of participants rated their understanding of genomics to be at a good level. Interestingly, when knowledge was measured more objectively using summative variable of KS, nurses in China (Wang et al. 2023) received the lowest scores which contrasts with their self‐rated knowledge and the nurses in the USA study (Gould and Seven 2022) actually achieved higher scores which matches their self‐rated knowledge. In the eight other studies, most nurses were uncertain of their competence, rating their own understanding lower than their KS indicated. An accurate understanding of one's own competence enhances confidence, which is essential for effective clinical tasks in genomics‐informed nursing (Cao et al. 2025). van der Giessen et al. (2020) found that self‐efficacy or confidence was low in communicating with patients with limited health literacy or a migrant background, although training was considered useful for improving these aspects. Calzone et al. (2026) concluded that nurses' capacity to integrate genomics into practice has remained both limited and largely unchanged over the past decade. Future research should examine factors that enhance nurses' confidence in genomics to support the translation of genomic knowledge into clinical practice.

In addition, a common finding across the studies was that nurses' genomic KS have remained relatively similar over the years. One interesting element was noted in two Turkish studies. Yeşilçinar et al. (2022a) study included nurses who were members of the Turkish Oncology Nursing Society and received better scores (9.36/12), while Ceylan et al.'s (2025) study, 3 years afterwards, included nurses working in two state hospitals in different specialty areas and reported lower knowledge scores (3.31/12). One reason might be that nurses working in oncology are more likely to be familiar with genomics through training or clinical experience, given that genomics applications have been most extensively developed and utilized in this field of medicine for over 20 years (Doroshow and Doroshow 2020). Conversely, such a conclusion cannot be substantiated based on the findings of a study in which no statistically significant difference was found in KS between general hospital nurses and cancer hospital nurses (Zhao et al. 2022). The cancer nurses only demonstrated a slightly higher recognition of the importance of genomics. Therefore, the area of clinical practice alone cannot be considered a sufficient explanation for the genomic competency level.

Efforts to build genomic knowledge and integrate it into nursing practice have been done globally. To strengthen knowledge of nurses, competency descriptions and guidelines for education have been established since the early 2000s in the United States of America (USA) (Calzone et al. 2024; Consensus Panel 2009), England (Kirk et al. 2014), and Italy (Kirk et al. 2011). In addition, they have been updated in the USA and England to reflect latest advancements (Calzone et al. 2024; National Health Services (NHS) 2023). Some nursing associations have provided comprehensive resources to support the integration of genomics into nursing education and practice. The American Association of Colleges of Nursing (2021) and the American Nurses Association (Consensus Panel 2009) have played a pivotal role in developing curricula guidelines to integrate genomics. In Canada, a national steering group, Canadian Nursing and Genomics, has been established for nurses to respond to advances in genomics (Canadian Nursing and Genomics 2024) and to support the development of nurses' genomic competence through an online toolkit (Limoges et al. 2025). In addition, locally originating projects have served as strong examples of efforts aimed at enhancing knowledge levels within a single university setting. In Finland, the PROFITU project started educational development for genomics‐informed nursing, piloted different methods of education, and established partnerships with experts, institutions, and industry leaders to integrate genomics into training (Halkoaho et al., n.d.; Laaksonen et al. 2022). GenoNurse is an example of an international cooperation and engagement project to pilot an educational model in Europe (Smolander et al. 2025). The international associations have also undertaken efforts to advocate for genomics‐informed nursing and accelerate implementation by organizing webinars and conferences and providing resources (Global Genomics Nursing Alliance (G2NA), n.d.; International Society of Nurses in Genetics (ISONG), n.d.). Nevertheless, the goal of preparing the nursing workforce for genomics has yet to be achieved (Calzone, Jenkins, et al. 2018; Calzone, Kirk, et al. 2018; Chow et al. 2023; McLaughlin et al. 2024; Saligan and Rivera 2014).

The awareness and knowledge capacity has been built in different parts of the globe, but more intensive collaboration is needed within nursing and between different disciplines locally and globally (Limoges et al. 2025). Such interorganizational networks have been shown to facilitate and strengthen implementation efforts (Sun et al. 2024). In addition, we suggest systematic interprofessional collaboration within healthcare organizations at various levels of management to drive integration forward (Yang et al. 2025). Leaders can utilize existing frameworks such as the roadmap (Tonkin et al. 2020a), the maturity matrix (Tonkin et al. 2020b), and the ACCESS framework (Katapodi et al. 2024) to systematically evaluate the state of knowledge and design strategies to enhance genomic literacy and clinical integration of genomic technologies (Limoges, Puddester, et al. 2024). Additionally, implementation research is needed to search and utilize more adaptive, scalable, and sustainable models for integrating genomics into practice, ideally creating frameworks that can be applied globally (Sun et al. 2024).

### 5.2 Importance of Genomics for Nurses

A central similarity across the included studies was nurses' recognition of the importance of genomic education, reported consistently in eight of the 10 studies. Given nurses' interest in genomics education, the discussion therefore focuses on identifying strategies that best support and enable their learning needs. This finding is consistent with previous literature (Calzone, Jenkins, et al. 2018; Calzone, Kirk, et al. 2018; Chow et al. 2023; McLaughlin et al. 2024; Saligan and Rivera 2014).

While most data in the included studies (Gould and Seven 2022; Limoges, Puddester, et al. 2024; Wang et al. 2023; Yeşilçinar et al. 2022a; Zhao et al. 2022) and other sources (Calzone et al. 2026; McLaughlin et al. 2024) indicate that nurses express intent to learn more about genomics, this comparative analysis revealed a modest decline in this intent over time. In addition, only in the studies conducted by Gould and Seven (2022) and Wang et al. (2023) did nurses' intention to pursue further genomics education match the level of importance they attributed to such training. Although nurses acknowledge the significance of genomics and education, and many express some intention to pursue further learning, nurses and midwives have been underrepresented among participants in formal genomic education courses in England (Carpenter‐Clawson et al. 2023). Future research should investigate the internal and external factors that either support or hinder participation in further education (Hakvoort et al. 2022; Vázquez‐Calatayud et al. 2021).

Addressing nurses' diverse needs in continuing education programs has shown to enhance engagement (Walter and Terry 2021). These needs, often shaped by factors such as years of professional experience (Hakvoort et al. 2022), can guide the development of educational opportunities. Involving nurses in the design of training programs and structuring the content to align with their needs is advisable. For instance, developing a modular training format could strengthen their intention to participate, as it allows individuals to select modules that are personally relevant and of interest. Nurses have indicated preferring genomics education through seminars, expert‐led question‐and‐answer sessions, and case‐based interactive computer modules, with nearly 75% favoring these formats (Hines‐Dowell et al. 2024). Flexible learning formats that accommodate practical constraints enable nurses to develop their genomic competency without compromising their clinical responsibilities (Cao et al. 2025).

Motivation is another factor that strengthens intention. In Kourkouta et al.'s (2021) article, the authors described several incentives that motivate nurses to participate in educational programs without specifying the content of the training. These included: voluntary participation; opportunities for exchanging perspectives and collaborative learning; the acquisition of new competencies; and a desire to enhance knowledge and skills. Nurses who have higher intrinsic motivation are more likely to enroll in further education (Sušilović et al. 2025).

Based on these findings, we propose involving nurses in the design of training programs and organizing the education into modules, allowing participants to select components that best suit their individual goals and that way motivate them more. Providing globally accessible educational resources in multiple languages may support the enhancement of nurses' knowledge. In addition, research focused on intention, motivation, and barriers to genomic training is important and should be prioritized.

### 5.3 Low Support Perception

In all articles that included data on managers or senior staff, nurses reported perceptions that genomics was not considered an important component of nursing by managerial or senior staff (Calzone et al. 2014; Ceylan et al. 2025; Gould and Seven 2022; Laaksonen dataset, 2025; Limoges, Puddester, et al. 2024; Wang et al. 2023; Yeşilçinar et al. 2022a; Zhao et al. 2022). Given nurses' low support perception, this part of the discussion is focused on strategies to better support nurses.

A lack of support from the managers and the senior staff could be a crucial factor that may affect genomic knowledge, confidence, and implementation of the genomics in practice (Limoges, Puddester, et al. 2024). The importance of leadership involvement and support for incorporating genomics into nursing practice is clearly emphasized in numerous articles (Calzone, Jenkins, et al. 2018; Chiu et al. 2024; Katapodi et al. 2024; Limoges, Halkoaho, et al. 2024). The role for nursing leaders could be to facilitate a positive attitude environment, enable nurses to participate in training to gain competence, influence motivation (Alsadaan et al. 2023), and monitor the development of genomic literacy of professionals in the healthcare environment (Tonkin et al. 2020a, 2020b).

Fostering cross‐disciplinary collaboration to detect emerging, practice‐changing research, developing strong nursing leadership to facilitate policy and workforce development, and creating a research framework to guide practice changes could be the next steps for nurse leaders (Limoges et al. 2025). Long‐term success in implementation requires support of leaders (Zebrowski et al. 2019) and encouragement of leaders as facilitators (Zebrowski et al. 2019), starting first with education of leaders (Limoges, Halkoaho, et al. 2024). Therefore, there is a need for developing education models for nurse leaders to better support nurses. Nurse leaders maintain an atmosphere where development and the incorporation of new evidence‐based innovations into care are valued. Therefore, they hold a crucial role in encouraging and empowering nurses to pursue training in genomics topics. A study conducted to assess the leadership team interventions to integrate genomics into clinical practice highlighted that improving knowledge and clinical implementation of genomics can be accomplished with leadership support (Calzone, Jenkins, et al. 2018). Collaboration is important to co‐design practice support. It considers validating nurses' practice concerns and understanding their knowledge needs and the types of questions patients are asking (Limoges, Puddester, et al. 2024). Such engagement ensures that practice support is both relevant and responsive to real‐world demands. The findings highlight an opportunity for global initiatives that support leadership development for the genomic era. Through global networking, both leaders and nurses can learn from their experiences and create solutions to their current challenges by sharing their experiences and perspectives. Therefore, attending international conferences, discussing current challenges, and drawing on diverse perspectives may help address clinical issues.

Possible solutions for the main study findings are presented in Table 5.

**TABLE 5:** Possible solutions for the main study findings.

Education Solutions	Co‐design training programs with nurses. Modular education for flexibility and motivation. Leadership‐focused education models for staff support.
Leadership Solutions	Systematic interprofessional collaboration across all management levels. Systematic use of existing frameworks (e.g., Roadmap, Maturity Matrix, ACCESS) to assess knowledge and plan strategies. Global collaboration to facilitate the exchange of experiences and best practices.
Research Solutions	Identify factors that strengthen nurses' confidence in genomics. Investigate intent, motivation, and barriers to genomic training. Conduct implementation research to create adaptive and sustainable integration models.

## 6 Limitations

This study had several limitations. First, some data were missing for certain variables (e.g., self‐rated knowledge) in few studies, which prevented their use in our analyses. Second, some of our key outcome variables were determined through nurses' voluntary self‐reports, which are subject to recall bias. Third, a formal quality assessment was not undertaken because the available studies reported mainly descriptive statistics (frequencies and percentages), and the aim of this secondary analysis was to examine cross‐country patterns of the data derived from a single, widely used instrument that was employed consistently across all included studies. Fourth, the study utilized both primary and secondary data. Although most of the data was secondary, a part of the Finnish dataset was primary and had not been published prior to this article. Fifth, the studies conducted with the participation of nurse educators and managers in addition to practicing nurses which may have influenced the mean knowledge score and limited its generalizability to practicing nurses only. Finally, cultural and institutional differences across settings may affect the generalizability of the results. Despite the limitations in study design, our findings allow comparison of key variables across different countries using a valid scale to assess genomic knowledge levels, needs, and perceptions of support among practicing nurses.

### 6.1 Implications

The incorporation of genomics is fundamental to nursing practice across all settings and countries. Once global challenges related to education, knowledge, and leadership support have been recognized, strengthening collaboration between nursing education and clinical practice becomes essential. As educational institutions begin to offer leadership training alongside bachelor‐level programmes, this may foster a trickle‐down effect in clinical settings, thereby supporting the implementation of genomics in nursing practice. Active involvement of nurses in genomics‐focused efforts may help improve their knowledge, identify existing gaps, and generate context‐specific solutions. To advance genomic competency in nursing, leadership engagement must move beyond general calls to action toward concrete strategies. A critical component is ensuring leaders receive foundational genomic education. This enables them to recognize staff training needs and provide appropriate support. We also recommend convening regional and international forums of key nursing leaders to develop collaborative approaches and actionable plans. Without this shift, progress is likely to remain limited. Global coordination and innovative interventions, such as the G2NA Roadmap (Global Genomics Nursing Alliance (G2NA), n.d.) and Assessment of Strategic Integration of Genomics Across Nursing (ASIGN) Maturity Matrix (Tonkin et al. 2020a) should guide these efforts (Limoges et al. 2025).

## 7 Conclusions

The key results of this comparative analysis reveal that nurses see genomics education as important, have moderate knowledge scores but low self‐rated knowledge rates, and perceive low support from their managers and senior staff. Based on the first two results, it can be concluded that nurses are critical or uncertain of their knowledge and therefore underprepared to deliver genomics‐informed practice. They experience conflict between the importance of genomics to nurses and not trusting their competence. The last result refers to the fact that nurses are not satisfied with the leadership support they receive. Importantly, these challenges are consistent across countries and remain unresolved over time, underscoring the need for innovative educational approaches and strengthened leadership support, and enhanced cross‐country collaboration to advance genomics integration in nursing practice. Future research should prioritize comparative analyses across countries to identify the factors most strongly supporting the implementation of genomics into clinical practice and to determine where the most substantial national‐level gains have been achieved.

### 7.1 Clinical Resources

In line with the identified gap between the perceived importance of genomics and nurses' knowledge, confidence, and organizational support, there is a clear need for accessible educational tools. The following examples of English‐language resources are presented to enhance genomic literacy and support genomics‐informed clinical practice.

- A toolkit for Canadian nurses: https://genomicstoolkit.my.canva.site/#home
- Genomics Education Programme (GEP): https://www.genomicseducation.hee.nhs.uk/
- Genomics Education Resource Center (GenomeEd): https://www.genome.gov/GenomeEd
- National Human Genome Research Institute About Genomics: https://www.genome.gov/about‐genomics.

## Funding

This research was supported in part by the Intramural Research Program of the National Institutes of Health (NIH) for K.C. No other external funding was received for this study.

## Conflicts of Interest

The authors declare no conflicts of interest.

## Supporting information




**Data S1:** Knowledge Score Items of GGNPS/GGNPS‐CA.

## Data Availability

The data that support the findings of this study are available from the corresponding author upon reasonable request.
