# Supplementary material for: Nurses' Genomic Knowledge, Attitudes, and Perceived Organizational Support: A Comparative Secondary Analysis of Genetics and Genomics in Nursing Practice Survey Data
Source: J Nurs Scholarsh. 2026 Jul 2;58(4):e70107. doi: 10.1111/jnu.70107 (PMC13329210; doi:10.1111/jnu.70107)
Supplement: Supplementary file 1 — Data S1: Knowledge Score Items of GGNPS/GGNPS‐CA. [file JNU-58-0-s001.docx]

| **Knowledge Score Items** |
| --- |
| 1. [P2-2a] A family history that includes only 1st degree relatives such as parents, siblings, and children should be taken on every new patient. *(Disagree)* |
| 1. [P2-2b] A family history that includes 2nd and 3rd degree relatives such as grandparents, aunts, uncles, and cousins should be taken for every new patient. *(Agree)* |
| 1. [P2-2c] Family history taking should be a key component of nursing care. *(Agree)* |
| 1. [P2-2d] There is a role for nurses in counseling patients about genetic risks. *(Agree)* |
| 1. [P4-1a] Do you think that genetic risk (e.g., as indicated by family history) has clinical relevance for breast cancer. *(Somewhat, A great deal)* |
| 1. [P4-1b] Do you think that genetic risk (e.g., as indicated by family history) has clinical relevance for colon cancer. *(Somewhat, A great deal)* |
| 1. [P4-1c] Do you think that genetic risk (e.g., as indicated by family history) has clinical relevance for coronary heart disease. *(Somewhat, A great deal)* |
| 1. [P4-1d] Do you think that genetic risk (e.g., as indicated by family history) has clinical relevance for diabetes. *(Somewhat, A great deal)* |
| 1. [P4-1f] Do you think that genetic risk (e.g., as indicated by family history) has clinical relevance for ovarian cancer. *(Somewhat, A great deal)* |
| 1. [P4-3b] ** Thinking about how you support clinical decisions … how important do you think each of the following is to consider?  *(Essential)* |
| 1. [P5-1] The DNA of sequences of two randomly selected healthy individuals of the same sex are 90-95% identical*. (False)* |
| 1. [P5-2] Most common diseases such as diabetes and heart disease are caused by a single gene variant. *(False)* |

Supplementary Data 1. Knowledge Score Items of GGNPS/GGNPS-CA

** P4-3b was changed in the Finnish version of GGNPS-CA to “Do you think that genetic risk (e.g., as indicated by family history) has clinical relevance for mental health?”
